# Supplementary material for: Identification of microRNAs That Provide a Low Light Stress Tolerance-Mediated Signaling Pathway during Vegetative Growth in Rice
Source: Plants (Basel). 2022 Sep 28;11(19):2558. doi: 10.3390/plants11192558 (PMC9614602; doi:10.3390/plants11192558)
Supplement: Supplementary file 1 [file plants-11-02558-s001.zip › Supplementary Figures.pdf]

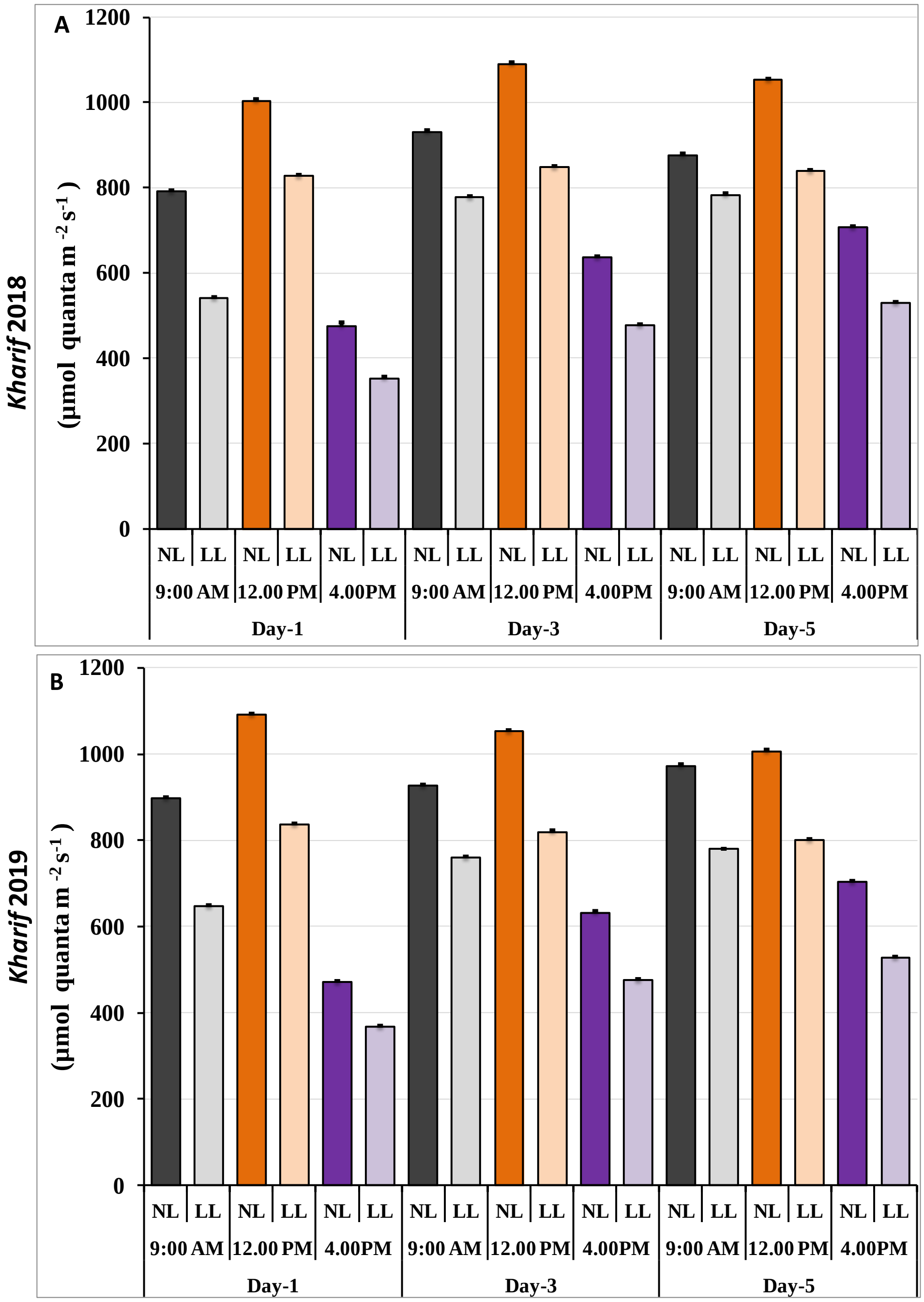

Figure S1. Spatiotemporal distribution of photosynthetic active radiation (PAR) above the canopy of tolerant and sensitive rice genotypes under normal (NL) and low light (LL) conditions in (A) *Kharif* season 2018, and (B) *Kharif* season 2019 (Data are means ± SD of n = 5 experiments).

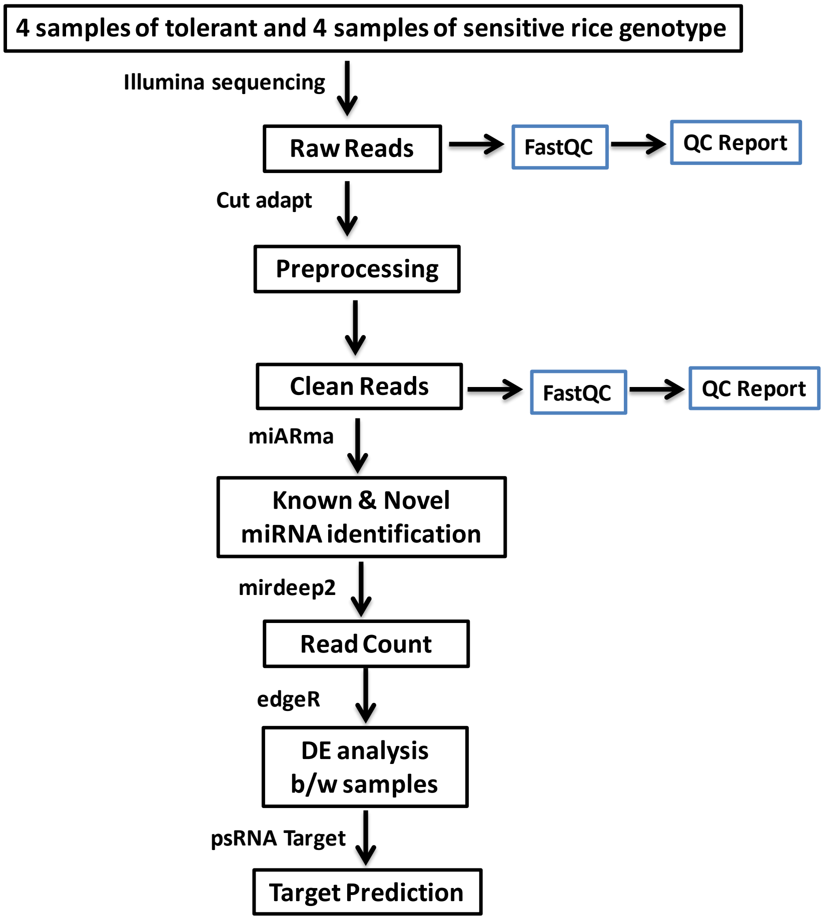

Figure S2. Work flow for Illumina sequencing and bioinformatics analysis for identification of miRNAs in low light treated and control samples of both the tolerant (Swarnaprabha) and sensitive (IR8) rice genotypes.

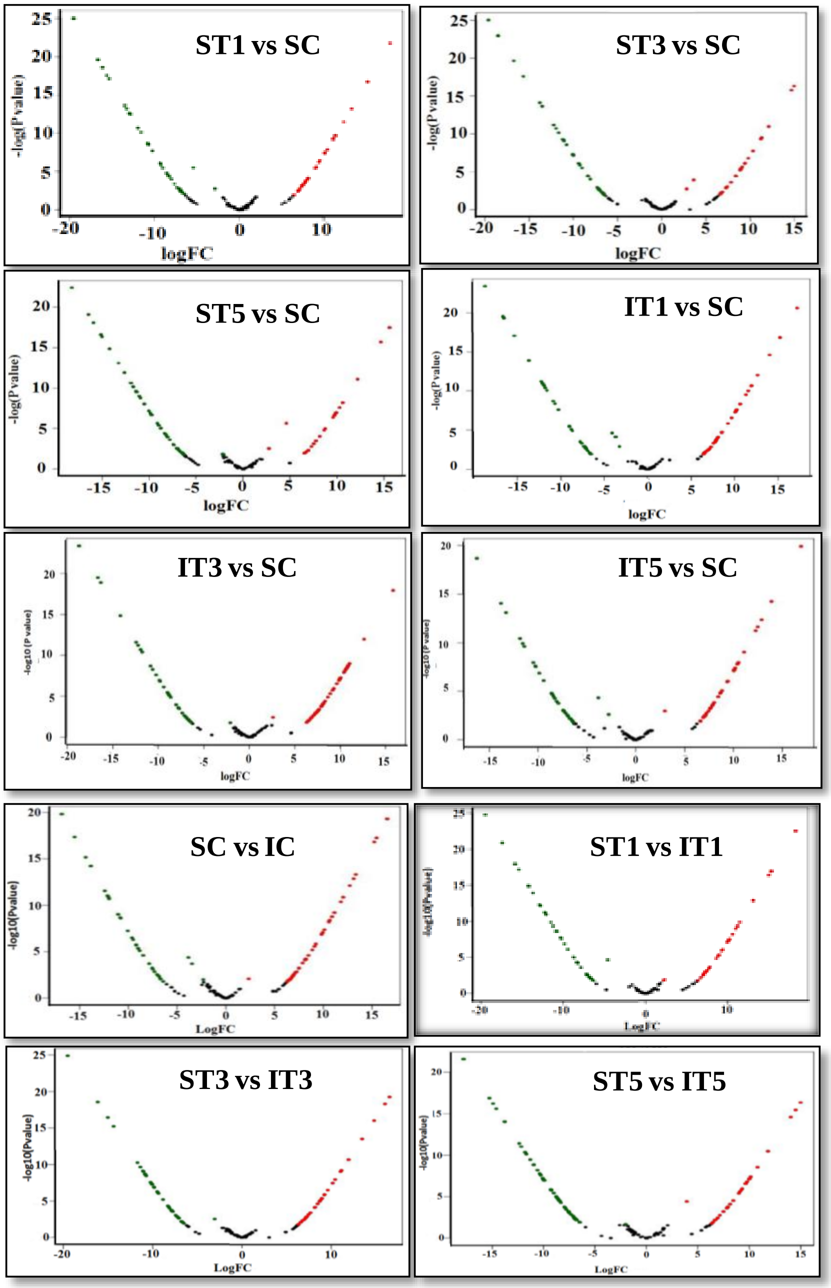

Figure S3. Volcano plot for differentially expressed miRNAs. X-axis shows the fold-change in miRNA expression between different samples, while Y-axis shows statistical significance of the differences. The comparison was in between treated and control samples of Swarnaprabha, and treated and control samples of IR8. Significantly up- and down-regulated miRNAs were filtered (|$\log_2$ (Fold Change)| > 1, Padj < 0.05) and highlighted in red and green dots, respectively. MiRNAs that are not differentially expressed are represented in black dots.

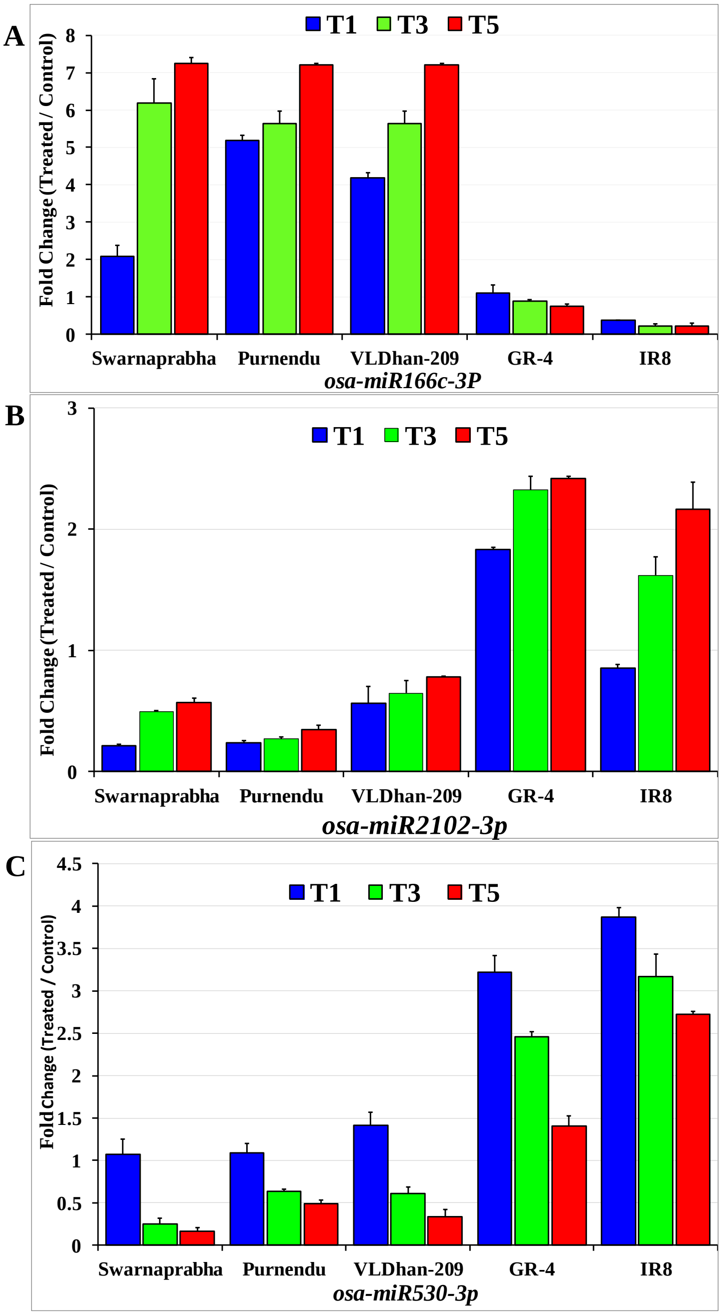

Figure S4 (A-C). Validation of three known differentially expressed miRNAs, *osa-miR166c-3p* (A), *osa-miR2102-3p* (B), and *osa-miR530-3p* (C) through miRNA specific qRT-PCR, identified from miRNA sequencing results of Swarnaprabha (SW) and IR8 after low light treatment with respect to control in three tolerant (SW, Purnendu, VLDhan-209) and two sensitive (GR4 and IR8) rice genotypes in *Kharif*, 2019. Each miRNA was amplified using mRQ 3′primer along with miRNA-specific and U6-specific 5′ primers. The primer sequences have been provided in Table S3. (# = miRNA-seq). Error bars are ±SD of the average of three miRNAs specific *q*RT-PCR replicates.

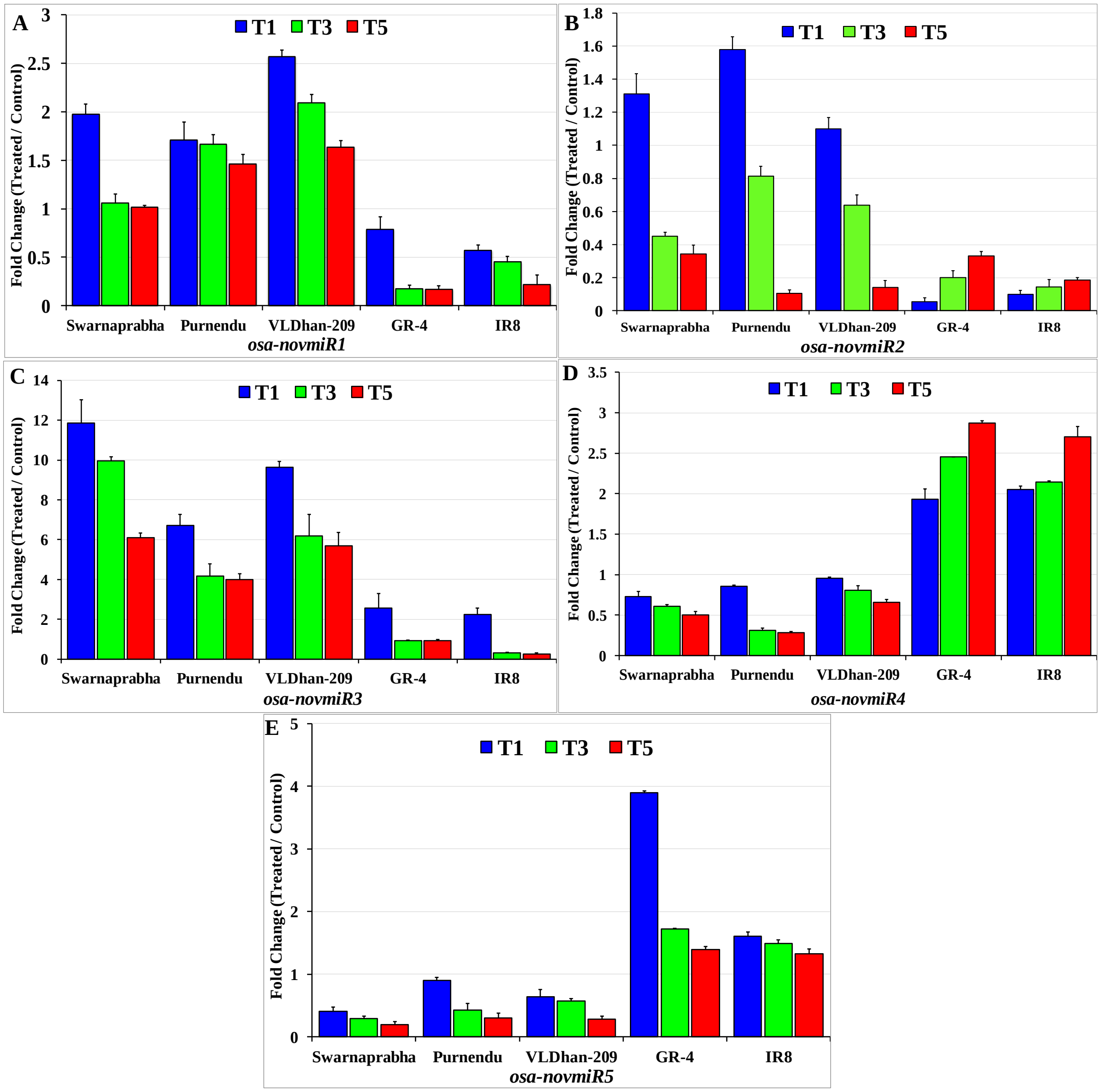

Figure S5 (A-E). Validation of five differentially expressed novel miRNAs, *osa-novmiR1*(A), *osa-novmiR2* (B), *osa-novmiR3*(C), *osa-novmiR4*(D), and *osa-novmiR5*(E), through miRNA specific qRT-PCR, identified from miRNA sequencing results of Swarnaprabha and IR8 after low light treatment with respect to control in three tolerant (Swarnaprabha, Purnendu, VLDhan-209) and two sensitive (GR4 and IR8) rice genotypes in *Kharif*, 2019. Each miRNA was amplified using mRQ 3′primer along with miRNA-specific and U6-specific 5′ primers. The primer sequences have been provided in Table S3. (# = miRNA-seq). Error bars are ±SD of the average of three miRNA specific *q*RT-PCR replicates.

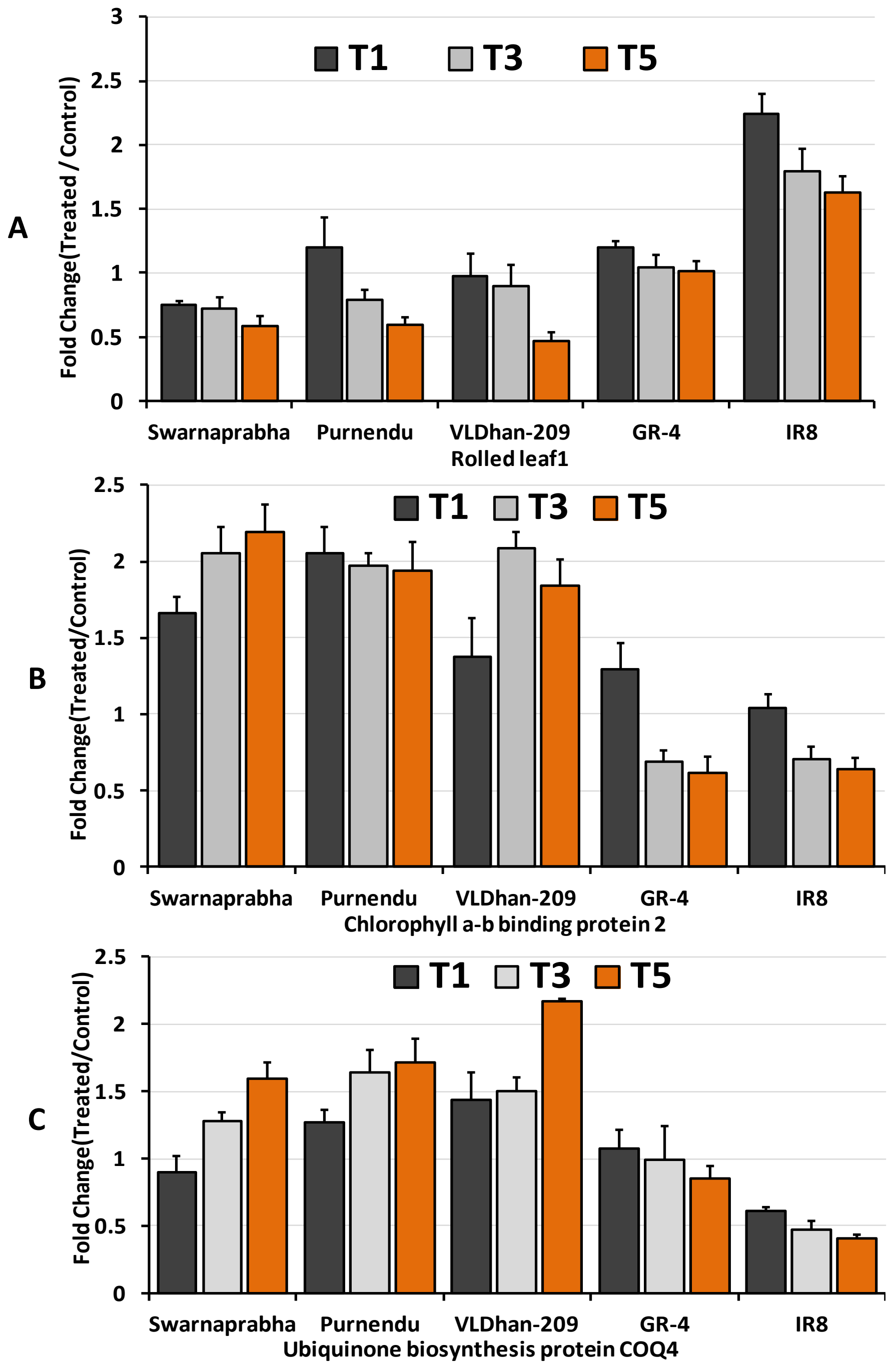

Figure S6 (A-C). Target gene expression analysis of the identified three known miRNAs in T1, T3 and T5 samples of three tolerant genotypes Swarnaprabha, Purnendu, VLDhan-209 and two sensitive rice genotypes GR4 and IR8 with respect to control. (A) Rolled leaf-1 expression, a target of miRNA *osa-miRNA166c-3P*, (B) Chlorophyll a-b binding protein, a target of miRNA *osa-miR2102-3p*, (C) Ubiquinone biosynthesis protein COQ4, a target of miRNA *osa-miR530-3p*. Each gene was amplified using gene-specific primers designed using Primer Blast Tool. Actin was taken as internal positive control. The primer sequences

have been provided in Table S3. Error bars are ±SD of the average of three *q*RT-PCR replicates.

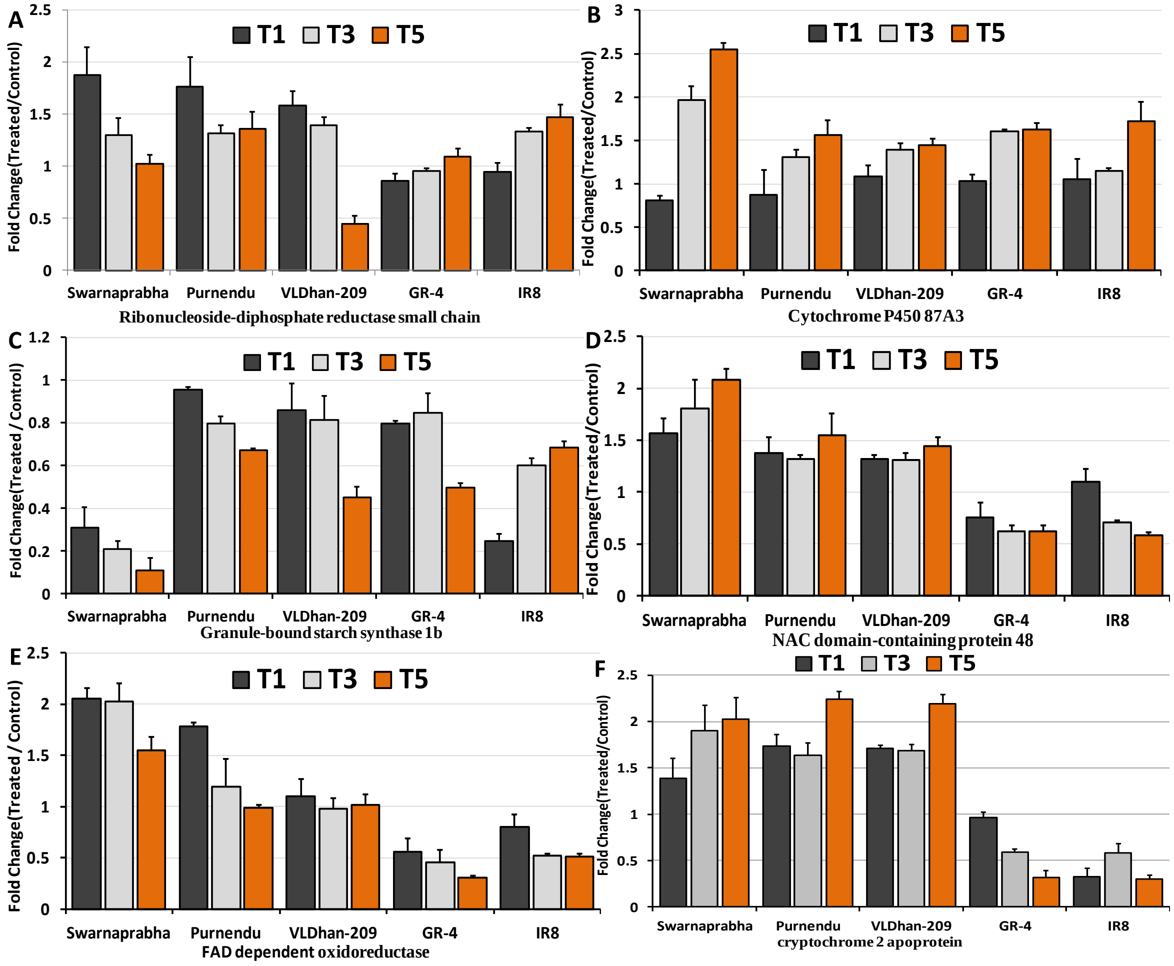

Figure S7 (A-F). Expression analysis of target genes of identified five novel miRNAs in T1, T3 and T5 sample of of three tolerant genotypes Swarnaprabha, Purnendu, VLDhan-209 and two sensitive rice genotypes GR4 and IR8 with respect to control. A) Ribonucleoside-diphosphate reductase expression, a target of miRNA *osa-novmiR1*, B) Cytochrome P450 87A3, a target of miRNA *osa-novmiR2*, C) *GBSS1b*, a target of miRNA *osa-novmiR3*, D) NAC domain- containing protein, a target of miRNA *osa-novmiR4*, E) FAD-dependent oxidoreductase, a target of miRNA *osa-novmiR5*, and F) Cryptochrome2 apoprotein, a target of miRNA *osa-novmiR5*. Each gene was amplified using gene-specific primers designed using Primer Blast. Actin was taken as internal positive control. The primer sequences have been provided in Table S3. Error bars are ±SD of the average of three *q*RT-PCR replicates.
